# The Functional Characterization of Epigenetically Related lncRNAs Involved in Dysregulated CeRNA–CeRNA Networks Across Eight Cancer Types

**DOI:** 10.3389/fcell.2021.649755

**Published:** 2021-06-17

**Authors:** Dahua Xu, Liqiang Wang, Sainan Pang, Meng Cao, Wenxiang Wang, Xiaorong Yu, Zhizhou Xu, Jiankai Xu, Hong Wang, Jianping Lu, Kongning Li

**Affiliations:** ^1^Key Laboratory of Tropical Translational Medicine of Ministry of Education, College of Biomedical Information and Engineering, Hainan Medical University, Haikou, China; ^2^College of Bioinformatics Science and Technology, Harbin Medical University, Harbin, China; ^3^Department of Thoracic Surgery, Harbin Medical University Cancer Hospital, Harbin, China

**Keywords:** pan-cancer, dysregulated ceRNA, epigenetically related lncRNA, diagnostic, prognosis

## Abstract

Numerous studies have demonstrated that lncRNAs could compete with other RNAs to bind miRNAs, as competing endogenous RNAs (ceRNAs), to regulate each other. On the other hand, ceRNAs were found to be recurrently dysregulated in cancer status. However, limited studies considered the upstream epigenetic regulatory factors that disrupted the normal competing mechanism. In the present study, we constructed the lncRNA-associated dysregulated ceRNA networks across eight cancer types. lncRNAs in the individual dysregulated network and pan-cancer core dysregulated ceRNA subnetwork were found to play more important roles than mRNAs. Integrating lncRNA methylation profiles, we identified 49 epigenetically related (ER) lncRNAs involved in the dysregulated ceRNA networks, including 18 epigenetically activated (EA) lncRNAs, 18 epigenetically silenced (ES) lncRNAs, and 13 rewired ER lncRNAs across eight cancer types. Furthermore, we evaluated the epigenetic regulating patterns of these lncRNAs and screened nine pan-cancer ER lncRNAs (six EA and three ES lncRNAs). The nine lncRNAs were found to regulate the cancer hallmarks by competing with mRNAs. Moreover, we found that integrating the expression and methylation profiles of the nine lncRNAs could predict cancer incidence in eight cancer types robustly and the cancer outcome of several cancer types. These results provide an improved understanding of methylation regulation to ceRNA and offer novel potential molecular therapeutic targets for the diagnosis and prognosis across different cancer types.

## Introduction

The competing endogenous RNA (ceRNA) network in tumor plays vital roles in the regulation of the biological function of pan-cancer. A growing number of researches have demonstrated that lncRNAs can act as endogenous molecular sponges to regulate the expression of mRNAs through communicating with miRNA response elements (Zhang G. et al., 2018; Qi et al., 2020; Wang W. et al., 2020). Further investigation of the ceRNA pairs in the dysregulated ceRNA network revealed more detailed biological functions related to the oncogenesis of malignant tumor (Yang et al., 2018; Zhang M. et al., 2020). Therefore, dysregulated ceRNA networks are involved in the key regulatory mechanism in the pathogenesis and development of cancer. However, the specific ceRNA dysregulated network in the pan-cancer remains to be elucidated.

As important ceRNA molecules of disease processes include cancer, lncRNAs have been implicated in biological, developmental, and pathological processes. Meanwhile, increasing evidences have indicated that DNA methylation is a key epigenetic signature implicated in the expression of lncRNAs. For instance, the alteration of DNA methylation status in the promoter region of lncRNA H19 during calcific aortic valve disease was associated with its upregulation (Hadji et al., 2016). In another case, Kumegawa et al. (2016) screened epigenetically silenced (ES) lncRNAs in colorectal cancer cells through a genome-wide analysis and found 20 dysregulated lncRNAs as targets of methylation. Moreover, the impact of DNA variation on the expression of lncRNA that influences ceRNA competition has been explored in a recent study. The aberrant promoter hypomethylation activated the lncRNA SNHG12, which leads to the upregulation of MAPK1 and E2F7 by binding to miR-129-5p in TMZ-resistant GBM cells and tissues (Lu et al., 2020). DNA methylation patterns implicated in the expression of protein-coding or non-coding transcripts across the pan-cancer were essential in the mechanisms of tumor development and cancer biology. However, the function of epigenetically related (ER) lncRNAs and the effect of lncRNAs alternations on relevant mRNAs in pan-cancer dysregulated ceRNA networks remain to be fully elucidated.

Here, we constructed the lncRNA-associated dysregulated ceRNA networks across eight cancer types by taking the advantage of RNA-sequencing and methylation data from TCGA (The Cancer Genome Atlas). We identified 49 ER lncRNAs involved in the dysregulated ceRNA networks. In addition, we excavated nine pan-cancer ER lncRNAs that regulate the cancer hallmarks [six epigenetically activated (EA) and three ES lncRNAs] through evaluating the epigenetic regulating patterns of these lncRNAs. Meanwhile, we found that these lncRNAs predict cancer incidence in eight cancer types robustly and predict the survival of these cancer patients by integrating molecular and clinical data. The findings are a coordinated effort to promote our understanding on regulatory mechanism of lncRNA-related ceRNA network governed by methylation in pan-cancer.

## Materials and Methods

### Transcriptome Expression Data Across Cancer Types

The gene and miRNA expression profiles were downloaded from The Cancer Genome Atlas (TCGA^1^) database release 10.0, which provided miRNASeq and HTSeq data. The lncRNA and mRNA annotation were downloaded from GENCODE (V22, GRCh38). Only tumor types with sufficient adjacent normal samples were considered (*N* > 30), including breast invasive carcinoma (BRCA), head and neck squamous cell carcinoma (HNSC), kidney renal clear cell carcinoma (KIRC), kidney renal papillary cell carcinoma (KIRP), liver hepatocellular carcinoma (LIHC), lung squamous cell carcinoma (LUSC), prostate adenocarcinoma (PRAD), and thyroid carcinoma (THCA) (Supplementary Table 1). The expressed genes (FPKM ≥ 1 in >70% samples) were selected for subsequent analyses. All of the expression profiles were log2 transformed.

### DNA Methylation Data Across Cancer Types

We also downloaded the HM450 DNA methylation profile of eight cancer types from TCGA (Supplementary Table 1). The probes with missing values in more than 30% of samples were removed, and other missing values were replaced by the mean value of the corresponding probe across samples.

### External Validation Data Across Cancer Types

The independent datasets were downloaded from the GEO database^2^, including 15 datasets acquired by the Affymetrix Human Genome U133 plus 2.0 array and Illumina HumanMethylation450 BeadChip across eight cancer types (Supplementary Table 2).

### miRNA–mRNA and miRNA–lncRNA Interaction Data

The experimental human miRNA–mRNA/lncRNA interactions were collected from four datasets, including miRTarBase 7.0 (Chou et al., 2018), miRecords 2013 (Xiao et al., 2009), starBase 2.0 (Li J. H et al., 2014), and lncRNASNP2 (Miao et al., 2018). Through redundancy analysis and standardization, 729,240 miRNA–mRNA pairs and 7092 miRNA–lncRNA pairs were obtained.

### Cancer Hallmarks, Cancer-Related mRNA, miRNA, and lncRNA Data

The cancer hallmark-associated GO terms were derived from a previous study (Plaisier et al., 2012). Cancer-related lncRNAs/mRNAs were collected from several databases, including COSMIC v89 (Forbes et al., 2015), NCG 6.0 (Repana et al., 2019), LncRNADisease (Chen et al., 2013), and lnc2Cancer v2.0 (Gao et al., 2019). Besides, we searched miRCancer (Xie et al., 2013) and used the eight caner types as keywords to filter cancer-related miRNA. In total, we obtained cancer-related mRNAs, miRNAs, and lncRNAs for 2362, 461, and 756 separately.

### Construction of the Dysregulated ceRNA Networks in Individual Cancer Type

**FIGURE 1:**
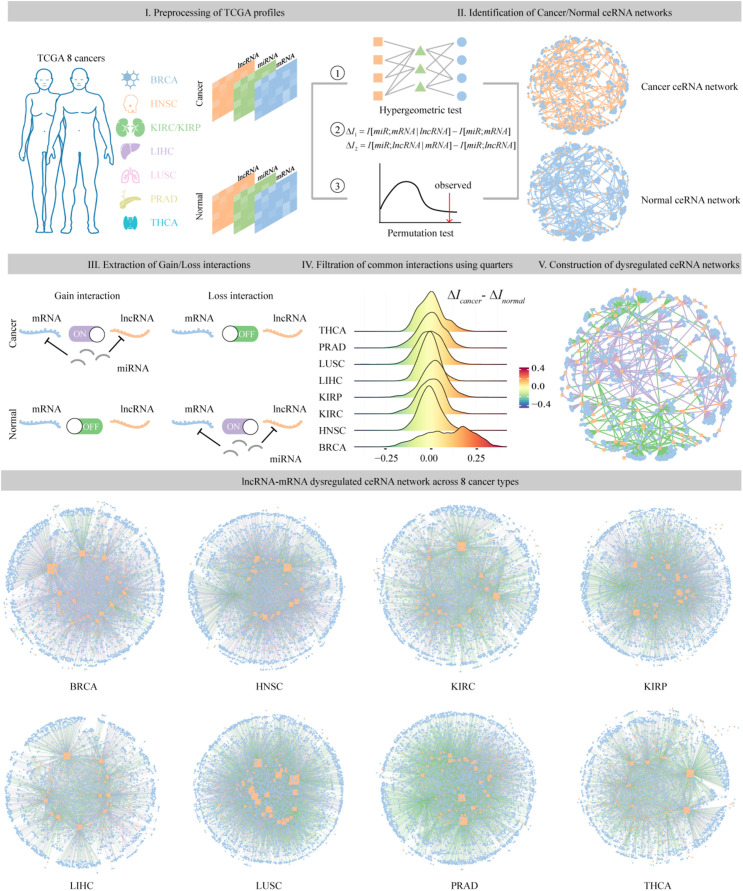
The pipeline of identification and construction of dysregulated ceRNA networks across eight cancer types. **Step I:** preprocessing of lncRNA, miRNA, and mRNA expression profiles from TCGA eight cancer types. **Step II:** identification of cancer and normal ceRNA networks using the method described in section “Materials and Methods.” **Step III:** extraction of ceRNA pairs that specifically existed in the cancer or normal samples. **Step IV:** filtration of common ceRNA pairs that occurred in both conditions using upper and lower quarters. **Step V:** assembling all the gain and loss interactions to construct dysregulated ceRNA networks.

To identify dysregulated ceRNA interactions, the miRNA-target regulations as well as expression associations among miRNA, lncRNA, and mRNA were considered. Then, we constructed the dysregulated ceRNA network according to the following three qualification rules (Figure 1).

#### Predicting Co-regulated Pairs

A hypergeometric test was used to compute the significance of shared miRNAs for each candidate lncRNA–mRNA pair. The *P*-value was calculated according to:

$$P=1-\sum_{i=0}^{r-1}\frac{\left(\begin{array}{c} K\\ i \end{array}\right)\left(\begin{array}{c} N-K\\ M-i \end{array}\right)}{\left(\begin{array}{c} N\\ M \end{array}\right)},$$

where *N* represents the total number of human miRNAs, *K* and *M* represent the total number of miRNAs targeting the mRNA and lncRNA, and *r* represents the number of common miRNAs between the lncRNA and mRNA. All *P*-values were subjected to Bonferroni correction, and co-regulated lncRNA–mRNA pairs with adjusted *P* < 0.01 were considered as candidate ceRNA interaction pairs.

#### Identification of ceRNA Interactions in Cancer and Normal Samples

Next, we developed a modified mutual information method based on *Hermes* (Sumazin et al., 2011) to identify ceRNA interactions in cancer and normal samples, respectively. First, we measured the competitive intensity between lncRNAs and mRNAs in cancer or normal conditions according to the following formula:

$$\begin{array}{c} \Delta I_{1}=I\left[miR;mRNA|lncRNA\right]-I\left[miR;mRNA\right]\\ \Delta I_{1}=I\left[miR;lncRNA|mRNA\right]-I\left[miR;lncRNA\right] \end{array}$$

In the formula, miR represents the miRNA set shared by lncRNAs and mRNAs. For each miRNA, *I*[*miR;mRNA*] is the mutual information between miRNA and mRNA, and *I*[*miR;mRNA| lncRNA*] is the mutual information between miRNA and mRNA under the lncRNA condition. Then, we randomly permuted the sample labels 100 times and compared the real Δ*I*_1_ with random values. We repeated the flow for Δ*I*_2_ and collected the *p*-values for each triplet. For each miR_*k*_ in the program, we converted the individual *p*-values, *p*_*k*1_ and *p*_*k*2_, to a χ^2^ test statistic using Fisher’s method:

$$X^{2}=-2\sum_{k=1}^{N}\ln\left(p_{k}\right)$$

where *N* is the total number of miRNAs in the program and *p*_*k*_ is the gather of *p*_*k*1_ and *p*_*k*2_. All the candidate ceRNA pairs with posteriori *p*-value < 0.01 and regulated by at least three common miRNAs were identified as ceRNA interactions in cancer or normal samples.

#### Construction of Dysregulated ceRNA Network

Finally, we reconstructed the dysregulated ceRNA network based on the acquired ceRNA interactions in cancer and normal samples. First, we defined the ceRNA pairs that specifically existed in cancer or normal samples as gain or loss interaction. Second, for each common interaction that occurred in both conditions, we computed the difference of Δ*I* in two status, defined as ΔΔ*I*. The pairs with ΔΔ*I* greater than 75% or less than 25% of all ΔΔ*I* values in a specific cancer type were identified as gain or loss interaction, while other common pairs were abandoned considering their similar competitive capacity in both status. Assembling all the gain and loss interactions, we finally obtained the cancer-related dysregulated ceRNA networks. In total, eight dysregulated ceRNA networks (DysCeNets) for eight cancer types were constructed.

### Identification of ER lncRNA in the Dysregulated ceRNA Network

Epigenetic regulation is one of the important mechanisms utilized to control lncRNA expression. To explore the association between lncRNA expression and methylation in the dysregulated ceRNA network, we identified ER lncRNA according to the method of Wang Z. et al. (2018). We first searched the probes in the promoter region of each lncRNA to acquire lncRNA–probe pairs. Then, Spearman correlation coefficient between the methylation and expression levels for each lncRNA–probe pair was calculated in each cancer type. The discrete categories included strongly negatively correlated (SNC, correlation coefficient: [−1, −0.5]), weakly negatively correlated (WNC, correlation coefficient: [−0.5, −0.25]), and no negative correlation (NNC, correlation coefficient: [−0.25, 1]), which were assigned based on the correlation coefficient. The probe with the highest coefficient was selected for the lncRNA if multiple probes were annotated to the same lncRNA promoter. Next, we defined lncRNA status according to the observed 30th and 70th beta values across tumor (T) and normal (N) samples. Then, we scored each lncRNA gene per cancer type according to the following rules:

(1) If percentile 70 < 0.25, the lncRNA was constitutively unmethylated in normal or tumor tissue; thus, we scored it as CUN or CUT.
(2) If percentile 30 > 0.75, the lncRNA was constitutively methylated in normal or tumor tissue; thus, we scored it as CMN or CMT.
(3) If percentile 30 > 0.25 and percentile 70 < 0.75, the lncRNA was intermediately methylated in normal or tumor tissue; thus, we score it as IMN or IMT.
(4) If it did not fall into any of the above categories, the lncRNA was variably methylated in normal or tumor tissue; thus, we score it as VMN or VMT.

Finally, we assigned a “call” for each of the possible combinations [3 (SNC, WNC, NNC) × 4 (CUN, CMN, VMN, IMN) × 4 (CUT, CMT, VMT, IMT)] per platform. In this way, the global trend of each lncRNA in one cancer type was acquired. Through combining the obtained pattern with the methylation level of this lncRNA in each cancer sample, we further determined the role of this lncRNA in a single sample. The epigenetic regulation types comprised EA and ES, and the other cases were not considered (Supplementary Table 3). According to the manually defined classifier, if the combination for the lncRNA and methylation probe was SNC × CMN × CUT and the beta value of the cancer sample at this probe was less than 0.25, we called the cancer sample EA at this probe or lncRNA. Next, epigenetic statuses of lncRNAs were characterized based on the percentage of regulated patients. If the number of EA samples was more than twice as the ES samples in a single cancer type, we determined it as EA lncRNA in this cancer type. Similarly, if the number of ES samples was more than twice as the EA samples in a single cancer type, we determined it as ES lncRNA in this cancer type. The others will be defined as multi-ER lncRNAs. The detailed information of these ER lncRNAs that occurred in a single cancer type is shown in Supplementary Table 4.

The characterizing process of ER lncRNAs in pan-cancer was similar as mentioned above. If the number of EA samples was more than twice as the ES samples in at least 75% of cancer types, we determined it as pan-cancer EA lncRNA. For example, the lncRNA was ER in four cancer types and the number of EA samples was twice greater than the ES sample in three cancer types; the percentage was 75%, and we considered this lncRNA as an EA lncRNA in pan-cancer. Next, if the number of ES samples was more than twice as the EA samples in at least 75% of cancer types, we determined it as an ES lncRNA in pan-cancer. The others will be defined as multi-ER lncRNAs, and these lncRNAs played different roles of epigenetic activation and epigenetic silencing in different cancer types. The detailed information of these ER lncRNAs that occurred in pan-cancer is shown in Supplementary Table 5.

### Development of ER lncRNA-Based Scores in Cancer Diagnosis

**FIGURE 2:**
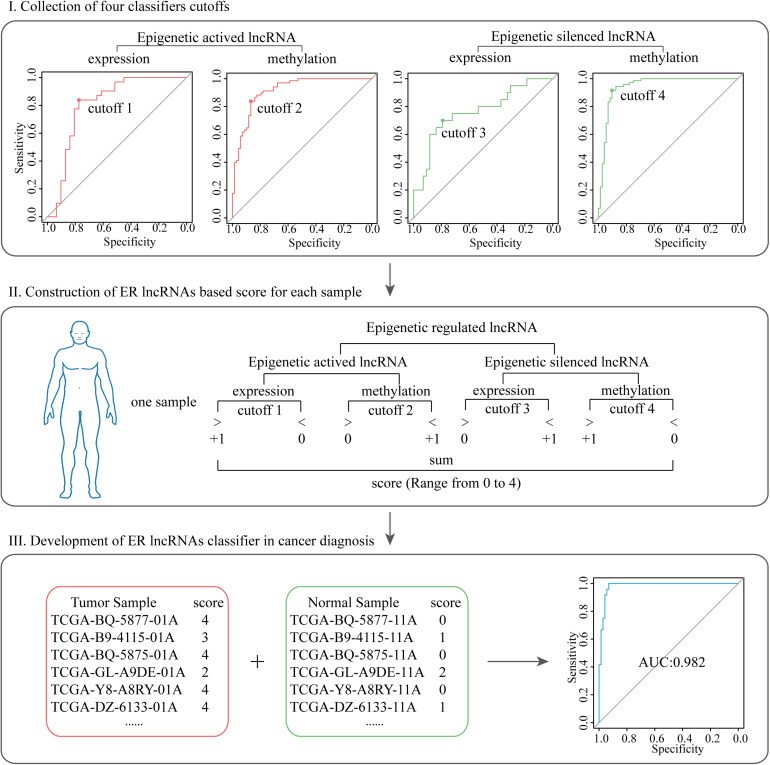
The pipeline of developing ER lncRNA-based scores in cancer diagnosis. **Step I:** collection of four classifier cutoffs based on the expression and methylation of EA and ES lncRNA, respectively. **Step II:** construction of ER lncRNA-based scores for each sample. **Step III:** development of ER lncRNA classifier in cancer diagnosis.

To evaluate the potential diagnosis capacity of ER lncRNAs, the scoring classifier was constructed using two-dimensional data (expression and methylation, Figure 2). We first build four classifiers that separated tumor and normal samples using the mean value of expression and methylation of EA and ES lncRNA, respectively. Next, the four cutoffs acquired from the abovementioned receiver operating characteristic (ROC) curve were collected, and an ER lncRNA-based score was constructed for each sample. We will assign a point when the sample meets either of the following situations: (1) the sample showed higher expression than the EA expression cutoff; (2) the sample showed lower methylation than the EA methylation cutoff; (3) the sample showed lower expression than the ES expression cutoff; (4) the sample showed higher methylation than the ES methylation cutoff. Then, we summarized these points for each sample of the ER lncRNA-based score (range from 0 to 4). A higher score denoted that the sample was subjected to epigenetic regulation in cancer. Finally, the scores of all tumor and normal samples in each cancer type were collected, and ROC curve analyses were conducted to investigate the diagnosis performance of the classifier.

### Survival Analysis

The Cox regression was performed to evaluate the prognosis of each ER lncRNA based on its expression or methylation level. Then, the ER lncRNAs with potential prognosis (cox-*P* < 0.3) were combined to obtain the survival score for each cancer sample according to the following formula:

$$S=\sum_{i=1}^{n}\frac{1-HR_{i}}{se\left(HR_{i}\right)}\times X_{i}$$

where *X*_*i*_ is the expression or methylation level of lncRNA *i* in sample *S*, *HR*_*i*_ is the overall hazard ratio of lncRNA *i*, *se*(*HR*_*i*_) is the standard estimates of *HR*_*i*_, and *n* is the number of ER lncRNAs.

The median score was used as the cutoff point to divide the patients into low-risk and high-risk groups. The overall survival (OS) of these groups was compared using log-rank test.

## Results

### Global Properties of Dysregulated ceRNA Networks Across Eight Cancer Types

**FIGURE 3:**
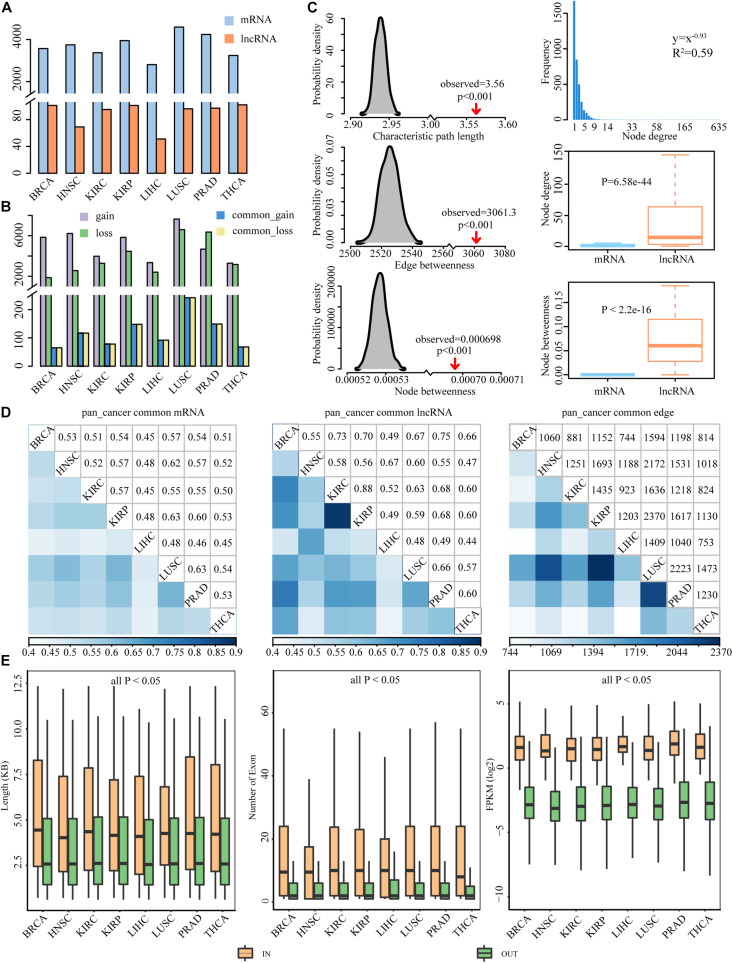
Property features of DysCeNets across eight cancer types. **(A)** The number of mRNA and lncRNA in DysCeNets. **(B)** The number of gain and loss interactions in DysCeNets. A purple column represents ceRNA pairs that specifically existed in cancer samples, a green column represents ceRNA pairs that specifically existed in normal samples, and blue and yellow columns represent upper and lower quarters of common ceRNA pairs. **(C)** The characteristic path length, average edge betweenness, and average node betweenness of DysCeNets compared to random networks are shown in the left panel. The distribution of node degree, the differences of node degree, and betweenness between mRNA and lncRNA are shown in the right panel. (Take BRCA as an example, the other cancer properties are shown in Supplementary Figure 1.) **(D)** The comparison of mRNAs, lncRNAs, and edges between any two cancer types. **(E)** The length, number of exons, and expression level of lncRNAs in the DysCeNets compared with lncRNAs not involved in the networks.

lncRNA has been found to act as ceRNA that indirectly regulates mRNA *via* shared miRNAs, and the dysregulation of the crosstalk between ceRNAs could promote the development of cancers (Zhang S. et al., 2018; Li P. et al., 2020). To assess dysregulated lncRNA-associated ceRNA patterns in cancer process, we identified the gain and loss ceRNA interactions and further constructed dysregulated ceRNA networks for eight cancer types (Figure 1). In total, we identified 6381 mRNAs and 154 lncRNAs participating in 47,714 dysregulated ceRNA interactions. In DysCeNets, there were 2807–4589 mRNA and 51–102 lncRNA involved in ceRNA dysregulation (Table 1 and Figure 3A). We then explored the distribution of gain and loss dysregulated patterns of ceRNA interaction across networks. As a result, there were 3360–7885 gain interactions and 1921–6846 loss interactions across eight cancer types (Figure 3B). These dysregulated ceRNA pairs were either specifically competing with miRNAs in cancer/normal context or significant differences in the intensity of competitive capacity between both statuses. These results suggest that ceRNA dysregulation was common in the cancer process.

**TABLE 1:** Statistics of nodes and edges in the ceRNA dysregulated network across eight cancer types.

	mRNA	lncRNA	gain_ special	loss_ special	gain_ common	loss_ common
BRCA	3570	101	5853	1856	65	65
HNSC	3750	69	6220	2557	117	117
KIRC	3373	95	3978	3280	78	78
KIRP	3948	101	5841	4464	148	148
LIHC	2807	51	3347	2398	92	92
LUSC	4589	96	7643	6604	242	242
PRAD	4241	97	4671	6366	149	149
THCA	3241	102	3292	3164	68	68

The global patterns of lncRNA-associated competing triplets and the characteristics of ceRNAs in the network across different cancer types have been revealed (Wang et al., 2015). However, few studies have focused on the dysregulated ceRNA interactions in pan-cancer. Through topological feature analysis, properties of DysCeNets were revealed (Figure 3C and Supplementary Figure 1). Firstly, the node degree distribution of the networks was investigated. We found that these DysCeNets revealed power-law distribution with *R*^2^ ranging from 0.57 to 0.62, suggesting that the networks displayed scale-free characteristics typical of biological networks. In each DysCeNet, most ceRNAs had few interacting dysregulated ceRNA partners, while a small subset of ceRNAs had a relatively large number of interacting dysregulated ceRNAs. In general, the characteristic path length, average node, and edge betweenness were significantly increased when compared with random networks (*P*-value < 0.001), implying that the DysCeNet had reduced global efficiency. In addition, we found that node degree and betweenness of lncRNAs were significantly higher than mRNAs (Wilcoxon test, *P*-value < 0.05), suggesting the leading roles of lncRNAs in the dysregulated networks.

Herein, we compared the attributes (including mRNAs, lncRNAs, and edges) of the DysCeNets and found that any two DysCeNet shared a large proportion of mRNA and lncRNA, implying that the lncRNA-associated ceRNA dysregulation was widespread in the cancer environment (Figure 3D). Besides, we found that the lncRNAs in the dysregulated ceRNA pairs were more consistent than mRNAs, suggesting that lncRNAs may play more crucial roles in ceRNA dysregulation. Moreover, DysCeNets obtained from similar original tissues tended to share more lncRNAs, which were consistent with previous studies (Xu et al., 2015; Zhang Y. et al., 2016). For instance, KIRC and KIRP are two types of kidney carcinomas, and approximately 88% of lncRNAs in KIRC also worked in KIRP. This analysis revealed that the molecular characterization of cancers with similar tissue of origin was more relevant than the others. Although there was a considerable mean of 1314 common pairs between any two cancer types, their Jaccard indexes ranged from 0.057 to 0.103, which were far less than the nodes’ indexes.

To explore the lncRNA properties as miRNA sponge in DysCeNets, the related characteristics including transcript length, number of exons, and expression level were compared to those lncRNAs that were not involved in DysCeNets. As a result, the lncRNAs in DysCeNets were found to have longer transcript length, own more exons, and express higher than other lncRNAs in eight cancer types (Wilcoxon test, *P*-value < 0.05, Figure 3E), suggesting that they are more adaptable to act as miRNA sponges. The detailed comparative information between lncRNA in and out networks was provided in Supplementary Table 6. Together, these results validated that lncRNAs are key components involved in ceRNA dysregulation.

### The Core Dysregulated ceRNA Component Is Strongly Related to Cancer Processes

**FIGURE 4:**
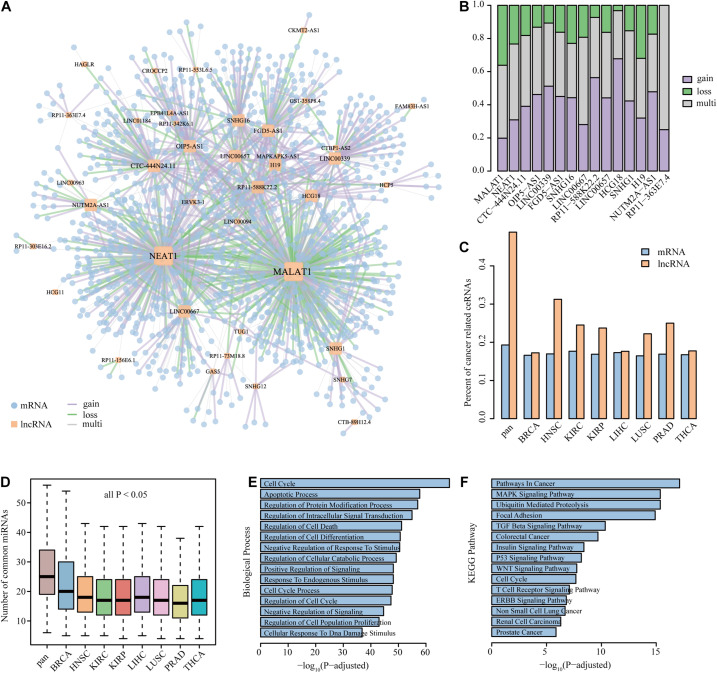
The property and functional analysis of the core component. **(A)** The largest component of the core dysregulated ceRNA interactions. **(B)** The property of edge linked to lncRNAs in the core component. The assignment of lncRNAs was sorted by node degree in the core component. Only show the lncRNAs with a degree of more than 10 in the core component. **(C**,**D)** The comparison of the percent of cancer-related ceRNAs and the number of common miRNAs between the core component ceRNAs and single dysregulated network. **(E**,**F)** The biological process and KEGG pathway enriched by the genes in the core component.

A common core of ceRNA regulatory interactions was defined as a component whose ceRNA triplets occurred in multiple cancer types. The core component could maintain the architecture of ceRNA networks across cancers and those ceRNAs in the component were found to be highly enriched in basic cellular processes to cancer (Xu et al., 2015). To determine the core component that exists in dysregulated ceRNA networks, we focused on the dysregulated ceRNA interactions that occurred in at least four cancer types. In total, 1713 edges were extracted to construct the core component, involving 1291 mRNAs and 43 lncRNAs (Figure 4A). We further defined the edges with consistent dysregulated type in more than 75% of cancers as gain or loss interactions and the others as multi-interactions in the pan-cancer dysregulated core component. We found that 57.62% of the edges in the core component showed the same dysregulated direction across multiple cancers. There were 590 gain interactions (59.78%) and 397 loss interactions (40.22%), indicating that a stable portion of RNA molecules tended to gain competitive relationships during cancer process. In addition, 42.38% of the edges showed different status among cancers, suggesting the complexity of ceRNA dysregulation.

Next, the proportion of different dysregulated interactions that lncRNAs linked in the core component is explored in Figure 4B. We found that lncRNAs with coincident property in the core component may associate with multiple cancer processes. For example, the lncRNA MALAT1, which owned the largest subnetwork and largest loss proportion, was found to regulate cancer glucose metabolism by enhancing mTOR-mediated translation of TCF7L2 in hepatocellular carcinoma (Malakar et al., 2019). Moreover, the lncRNA MALAT1 could mediate cisplatin resistance *via* the miR-101-3p/VEGF-C pathway in bladder cancer (Liu et al., 2019) and promote cell proliferation and inhibit apoptosis by sponging miR-101 in colorectal cancer (Si et al., 2019). Another case is the lncRNA HCG18, possessing the most gain interactions, which could cooperate with NOTCH1 to regulate the proliferation and migration of bladder cancer cells (Xu et al., 2019). In addition, HCG18 was identified as an immune-related signature and showed prognostic efficacy for anaplastic gliomas (Wang W. et al., 2018). The NEAT1–MET axis was identified as gain interaction in our pan-cancer core component, suggesting that the competitive relation between NEAT1 and MET happened in cancer environment. Several studies have proved that NEAT1 can regulate c-met *via* ceRNA mechanism in different cancer types. For instance, NEAT1 suppressed sorafenib sensitivity of hepatocellular carcinoma cells *via* regulating miR-335/c-Met (Chen and Xia, 2019). NEAT1 was also found to regulate the growth, invasion, and migration of pancreatic cancer cells through microRNA-335-5p/c-met (Cao et al., 2016). These results further verified the validity of our study, and the utilization of the method could provide potential cancer biomarkers.

Using a cohort of publicly available datasets including COSMIC, NCG, LncRNADisease, and lnc2Cancer, cancer-related mRNAs and lncRNAs were collected. We found that each DysCeNet owned 17.24–18.47% cancer-related mRNAs and 28.43–42.03% cancer-related lncRNAs. Through comparing the core component with single DysCeNet, we found that the percent of cancer-related ceRNAs (especially lncRNAs) in the core component was higher than that in single dysregulated network (Figure 4C). The large proportion of cancer-related lncRNAs in dysregulated networks and core component further confirmed the crucial position of lncRNAs, which was consistent with previous results. A previous study has demonstrated the relationship between the number of common miRNAs and the intensity of ceRNA competitive capacity; co-expression of ceRNAs in the network could increase with the number of common miRNAs (Xu et al., 2015). In our study, we found that the numbers of common and cancer-related miRNAs that ceRNAs compete with in the core component were significantly increased than those in a single dysregulated network (Wilcoxon test, all *P*-value < 0.05, Figure 4D and Supplementary Figure 2). This result revealed that the stable ceRNA pairs were inclined to dysregulate in the pan-cancer level. Due to the limitation of annotated information for lncRNAs, lncRNA functions were frequently presumed based on known functions of related mRNAs (Wang et al., 2015, 2019; Song et al., 2017). Thus, the biological process and KEGG pathway enrichments were tested using mRNAs that occurred in the component. Processes for cell proliferation (such as cell death and cell cycle) and cancer-related pathways (such as TGF-beta signaling pathway, MAPK signaling pathway, and pathways in cancer) were highly enriched (Figures 4E,F). Overall, these observations suggest that the core dysregulated ceRNA component was strongly related to cancer processes and further proved the importance of lncRNAs.

### Identification of ER lncRNAs Involved in ceRNA Dysregulation

**FIGURE 5:**
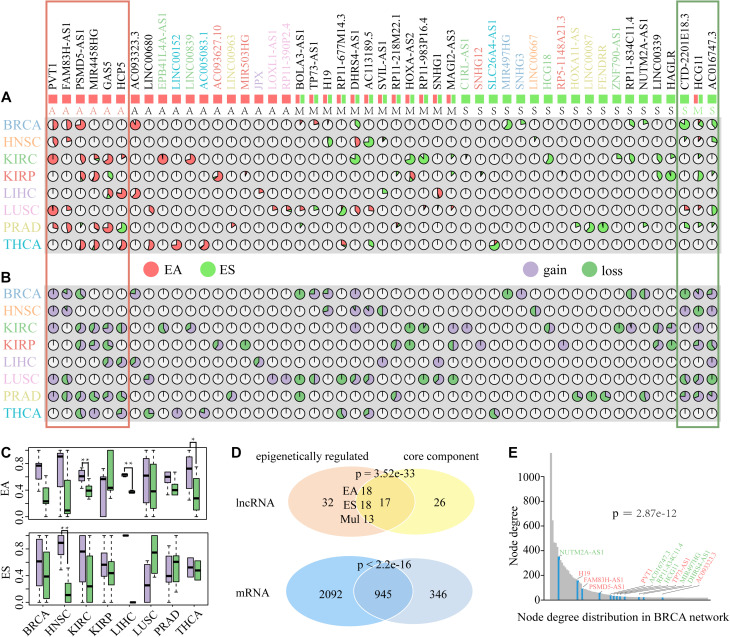
The distribution and attribute of ER lncRNAs in DysCeNets. **(A)** Percentages of EA and ES lncRNAs in eight cancer types. Each pie chart indicates the percentage of each lncRNA epigenetic alteration in each cancer type. Red indicates EA lncRNAs, and green indicates ES lncRNAs. The symbol of lncRNAs with special color represents the lncRNA altered in a specific cancer type. Symbols below with A represented EA lncRNAs, S represented ES lncRNAs, and M represented multi-ER lncRNAs. The red frame represents the lncRNAs epigenetically activated in pan-cancer, and the green frame represents the lncRNAs epigenetically silenced in pan-cancer. **(B)** The property of edges linked to ER lncRNAs in DysCeNets. Each pie chart indicates the percentage of dysregulated status. Purple indicates gain interaction, and green indicates loss interaction. **(C)** The property of edge linked to EA and ES lncRNAs in DysCeNets. **P-value < 0.05, *P-value < 0.1. **(D)** The comparison of lncRNAs and related mRNAs between epigenetically related ceRNAs and core component. **(E)** The node degree distribution of top 100 ceRNAs in BRCA DysCeNet. The blue column represents the node degree of ER lncRNAs. The symbol colored red represents EA lncRNAs, and the symbol colored green represents ES lncRNAs.

Growing evidences suggest that DNA methylation, a fundamental feature of epigenomes, can affect lncRNA expression, and there are intricate regulatory relationships between DNA methylation and lncRNA (Hadji et al., 2016; Yang et al., 2017; Zhi et al., 2018). Among these studies, Wang et al. characterized the epigenetic landscape of lncRNAs and identified recurrent ER lncRNAs in 20 cancer types (Wang Z. et al., 2018). However, the function of ER lncRNAs and the effect of lncRNA alternations on relevant mRNAs have not yet been explored. Here, we combined expression and methylation data to identify ER lncRNA involved in a single-ceRNA dysregulated network. All ER lncRNAs showed a negative correlation between their expression and promoter DNA methylation status. For EA lncRNAs, they showed hypermethylation and low expression in normal samples, while their methylation level decreased and expression was upregulated in tumor samples. For ES lncRNAs, they showed hypomethylation and high expression in normal samples, while their methylation level increased and expression was downregulated in tumor samples. Based on these principles, we totally identified 49 ER lncRNAs with a rate of 31.82% (154 lncRNAs in total) involved in DysCeNets. Through analyzing the epigenetic status of each ER lncRNA in the different samples of single cancer type, it was found that the vast majority of ER lncRNAs in single cancer were either inclined to EA (EA/ES > 2) or ES (ES/EA > 2) (Figure 5A and Supplementary Table 4). Only SVIL-AS1 in HNSC and RP11-218M22.1 in PRAD were subjected to complex regulation. These results revealed that the epigenetic regulation of lncRNAs showed a tendency in a single cancer type. Next, we explored the role of each ER lncRNA across cancer types and found that there were 22 ER lncRNAs that occurred in unique cancer type (10 EA and 12 ES lncRNAs), while 27 ER lncRNAs were regulated by DNA methylation in multiple carcinomas (Figure 5A). We further investigated the regulatory tendency of ER lncRNAs in pan-cancer based on the status of ER lncRNAs in each cancer type. For ER lncRNAs that occurred in pan-cancer, we identified consistently EA and ES lncRNA in pan-cancer (at least 75% of cancer types), including eight EA lncRNAs and six ES lncRNAs. In addition, the function of some ER lncRNAs in different cancer types was rewired. We recorded these lncRNAs as multi-ER lncRNAs based on their epigenetic regulation across cancer types. In summary, there were 18 EA lncRNAs, 18 ES lncRNAs, and 13 multi-ER lncRNAs involved in DysCeNets. From the landscape of lncRNAs, we could clearly know their epigenetic status in different cancer types. For instance, lncRNA PVT1 was EA in BRCA, HNSC, KIRC, LUSC, and PRAD cancer types, while lncRNA HCG18 was ES in only KIRC carcinoma.

To explore the effects of ER lncRNAs on ceRNA dysregulation, we next characterized the proportion of edges linked to ER lncRNAs. As shown in Figure 5B, a large scale of EA and ES lncRNAs were inclined to possess gain interaction while a small part of lncRNAs linked with loss interaction in each DysCeNet. We further compared the number of gain and loss interactions in which EA or ES lncRNAs regulated. The result showed that EA lncRNAs in KIRC, LIHC, and THCA DysCeNets owned more gain interactions than loss interactions, and ES lncRNAs in HNSC DysCeNet had the same phenomenon (Figure 5C and Supplementary Figure 3). Together, these results indicated that EA and ES lncRNAs were inclined to possess gain interaction in most cancer types (other cancers showed similar tendency but their *P*-values were not significant, which may be due to the limited number of ER lncRNAs in comparative groups). In addition, 17 ER lncRNAs and 945 related mRNAs were highly enriched in the core dysregulated ceRNA component above (Hypergeometric test, Figure 5D), which implied the vital function of ER lncRNAs in common cancer processes. Notably, the ER lncRNAs were also highly enriched in the top 100 nodes with the largest degree in each DysCeNet (Fisher test, Supplementary Figure 4). For example, 12 of 15 ER lncRNAs in BRCA DysCeNet were included in the top 100 of 3671 nodes (*P*-value = 2.87e−12, 12 of 15 vs. 100 of 3671, Fisher test, Figure 5E). All these observations suggest that the ER lncRNA might influence the stability of ceRNA interactions and further affect the cancer process.

### Identification of Potential Diagnostic ER lncRNAs

**FIGURE 6:**
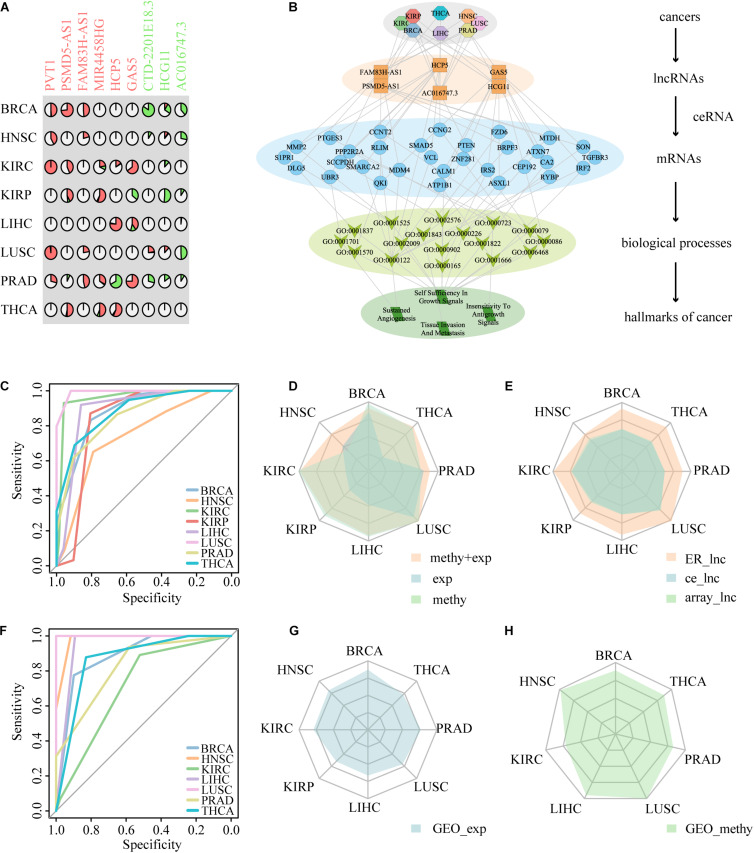
The performance of nine ER lncRNAs in distinguishing cancer patients from normal samples. **(A)** The layout of nine ER lncRNAs. The symbol of lncRNAs with red color represents the lncRNA epigenetically activated in pan-cancer, and the symbol of lncRNAs with green color represents the lncRNA epigenetically silenced in pan-cancer. **(B)** Summary of the hierarchical model to systematically understand the function of ER lncRNAs in DysCeNets. The model is laid out hierarchically with (from the top down) cancers, ER lncRNAs, mRNAs, annotated GO biological process terms, and hallmarks of cancers. **(C)** The ROC curve of ER lncRNAs based on two-dimensional datasets. **(D)** The performance of ER lncRNAs classifiers based on TCGA expression, TCGA methylation, and expression combined methylation levels. **(E)** The comparison of the classifiers among nine ER lncRNAs, nine lncRNAs randomly selected from DysCeNets, and nine lncRNAs randomly selected from expression/methylation profiles. **(F)** The ROC curve of ER lncRNAs based on the GEO methylation datasets. **(G**,**H)** The validated ER lncRNAs classifiers using external expression and methylation GEO datasets.

Epigenetic alterations have been established as one of the hallmarks of tumorigenesis, and the ER lncRNAs may provide new insight into the cancer diagnosis. We first filtrated ER lncRNAs with continuous status in multiple cancers (EA samples/ES samples > 2 or <0.5 in at least three cancer types) and obtained six EA lncRNAs (PVT1, PSMD5-AS1, FAM83H-AS1, MIR4458HG, HCP5, and GAS5) and three ES lncRNAs (CTD-2201E18.3, HCG11, and AC016747.3) (Figure 6A and Table 2). The association between expression and DNA methylation of these lncRNAs has been revealed in several studies. For instance, the EA lncRNA PVT1 expression was strongly and negatively correlated with its methylation status in uveal melanoma (Xu et al., 2017). Hypomethylation within another EA lncRNA HCP5 involves a CpG site that contains a single-nucleotide polymorphism in linkage disequilibrium with HLA-B^∗^27 and that controls DNA methylation at this locus in an allele-specific manner in ankylosing spondylitis (Coit et al., 2019). Overall, these pan-cancer ER lncRNAs were associated with multiple complex diseases. Next, we developed a frame to understand the relation between ER lncRNAs and cancers by connecting ER lncRNAs with cancer hallmark associated GO terms derived from a previous study (Plaisier et al., 2012). In the hierarchical model, the ER lncRNAs were firstly linked to mRNAs through ceRNA dysregulation. Then, ER lncRNA-related mRNAs were associated with biological processes and finally connected to cancer hallmarks. Through mapping hallmark genes to the ER lncRNAs, four cancer hallmarks including sustained angiogenesis, self-sufficiency in growth signals, insensitivity to antigrowth signals, and tissue invasion and metastasis were found to be associated with six ER lncRNAs in eight cancer types (Figure 6B). These results can help us comprehend how pan-cancer ER lncRNAs regulate mRNAs through ceRNA dysregulation and further influence cancer biological processes.

**TABLE 2:** Detailed information of nine epigenetically related lncRNAs.

Ensemble ID	Gene symbol	ER type	Cancer type
ENSG00000249859	PVT1	EA	BRCA, HNSC, KIRC, LUSC, PRAD
ENSG00000226752	PSMD5-AS1	EA	BRCA, KIRC, KIRP, THCA
ENSG00000203499	FAM83H-AS1	EA	BRCA, HNSC, LUSC, PRAD
ENSG00000247516	MIR4458HG	EA	KIRC, KIRP, PRAD, THCA
ENSG00000206337	HCP5	EA	KIRC, LIHC, THCA
ENSG00000234741	GAS5	EA	KIRC, LIHC, PRAD
ENSG00000212978	CTD-2201E18.3	ES	BRCA, HNSC, KIRP, LIHC, LUSC, PRAD
ENSG00000228223	HCG11	ES	BRCA, KIRC, KIRP, PRAD
ENSG00000177738	AC016747.3	ES	BRCA, HNSC, PRAD

Cancer-related genes usually showed significant differences in cancer and normal tissues and thus could distinguish carcinoma and normal samples as biomarkers (Smolle et al., 2019; Yoon et al., 2020). We have previously discovered the important roles of ER lncRNAs in DysCeNet and their guidance on the dysregulation of ceRNA interactions. Next, we expected to predict the status of cancer through these important nodes involved in DysCeNets. To determine whether these ER lncRNAs have the diagnostic capacity in multiple cancer types, we developed a classifier based on these ER lncRNAs as described in section “Materials and Methods.” This method systematically integrated the methylation and expression levels of ER lncRNAs. If the EA lncRNAs showed hypomethylation and high expression and the ES lncRNAs showed hypermethylation and low expression in one sample (based on four cutoffs obtained by ROC curves), we would assign the maximal score to that sample (scores of samples ranged from 0 to 4). Based on the scoring principle, the cancer samples with ER status would get higher scores, while the normal samples would hold lower scores. To verify the predictive validity of the score, we calculated the areas under the curve (AUCs) of our method and found that the AUCs distributed in 0.7412–0.9917 across eight cancer types (Figure 6C). We then compared the AUCs of our method in eight cancer types with the method that simply considered methylation or expression level of the same ER lncRNAs. As shown in Figure 6D and Supplementary Table 7, the classifier performed better when two-dimensional data instead of single-platform data were considered, and the single methylation dimension-based classifier performed apparently better than the single expression dimension-based classifier. Furthermore, we randomly selected nine lncRNAs from DysCeNets and expression/methylation profiles and conducted two classifiers using our method based on these random lncRNAs for 1000 times. We found that the performance of nine ER signatures across eight cancer types was significantly higher than those nine lncRNAs randomly selected (Figure 6E). These results proved the validity of our classifier based on two-dimension data in distinguishing cancer and normal samples. To further test the predictive effect of these nine ER lncRNAs on cancer, we downloaded 15 sets of independent data from the GEO database. Due to the lack of a complete omics study like TCGA, we separately obtained eight sets of expression profiles and seven sets of methylation profiles (lacking the KIRP methylation dataset). Using these 15 external GEO datasets, we developed two classifiers that simply considered methylation or expression level of our pan-cancer ER lncRNAs as before. The AUCs of the classifiers based on the external methylation data of these ER lncRNAs ranged from 0.7124 to 1 (Figure 6F). Similar to the result of TCGA datasets, the lncRNA methylation status separated the tumor and normal sample better than expression data (Figures 6G,H). In conclusion, these nine ER lncRNAs could serve as predictive biomarkers for multiple cancers. Moreover, the prediction effect of ER lncRNAs would reach best when combining expression and methylation data. For cases of lacking paired omics data, it is better to utilize methylation level data than expression level data to construct the classifier.

### The ER lncRNAs Predict the Prognosis of Cancer Patients

**FIGURE 7:**
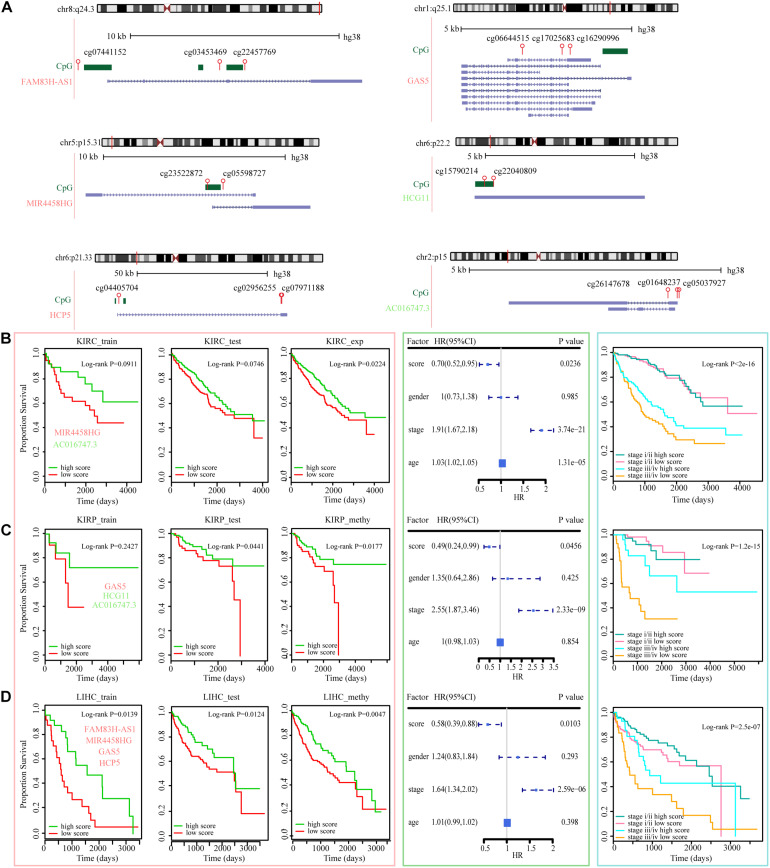
ER lncRNAs as prognostic factors in various tumors. **(A)** The locations of ER lncRNAs occurred in three datasets (blue), CpG islands (green), and HM450 probes (red) in GRCh38 reference human genome. The symbol colored red represents EA lncRNAs, and the symbol colored green represents ES lncRNAs. **(B**–**D)** Kaplan–Meier estimates of OS of patients with KIRC expression, KIRP methylation, and LIHC methylation levels according to the ER lncRNA signature. Kaplan–Meier estimates of the OS of patients in train, test, and all sets according to the ER lncRNAs are shown in the left panel. The hazard ratios of clinical factors using a multivariate Cox regression model are shown in the middle panel. Kaplan–Meier estimates of the OS between low-stage and high-stage patients according to the ER lncRNAs are shown in the right panel.

The ability of ER lncRNAs to cancer diagnosis has been examined herein before. We then evaluated the effect of these ER lncRNAs on cancer progression, that is, to determine whether patients with different OS could be distinguished based on expression or methylation data of these ER lncRNAs. As described in section “Materials and Methods,” the TCGA paired samples were regarded as a train set, and other TCGA samples were regarded as the test set. We first estimated the survival difference between high and low score groups in the train set and then validated the effectiveness of these ER lncRNAs in the test set using the parameters [including median score, HR, and se(HR)] of the train set. The low survival score based on ER lncRNAs predicted poor prognosis in most cancer train sets, and there were three test sets that performed well, including KIRC expression dataset, KIRP, and LIHC methylation datasets. The genome information and related CpG probes of the ER lncRNAs that occurred in three datasets are shown in Figure 7A. There are six ER lncRNAs whose methylation levels in the promoter region changed, thus affecting lncRNA expression in these three datasets, including four EA lncRNAs (FAM83H-AS1, MIR4458HG, HCP5, and GAS5) and two ES lncRNAs (HCG11 and AC016747.3). In particular, FAM83H-AS1, HCP5, GAS5, and HCG11 were identified in the pan-cancer core component mentioned above. As shown in the left panel of Figures 7B–D, the survival score based on ER lncRNAs could predict the prognosis of cancer patients. Next, the multivariate Cox regression model was used to verify the prognostic efficacy of multiple clinical factors. The survival score based on ER lncRNAs was found to be positive as a protective factor, and the stage conversely showed prognostic efficacy as a risk factor (all *P*-value < 0.05, Figures 7B–D, middle panel). Then, patients with stage I and II were merged into the low-stage group, and patients with stage III and IV were merged into the high-stage group. We integrated the score based on ER lncRNA and stage information and estimated the survival curves by the similar method above. It was found that the prognosis capacity was stronger than the method using molecular-level data alone. In particular, in the case of the high-stage group, the OS of patients with high scores showed significantly better than those with low scores (Figures 7B–D, right panel and Supplementary Figure 5). These results implied that the combination of molecular and clinical data could better predict the survival of these cancer patients. Collectively, the data suggest that ER lncRNAs involved in ceRNA dysregulated network could not only act as cancer diagnostic markers but also influence cancer progression and outcome in some cancer types.

## Discussion

The dysregulation of ceRNA was both widespread and influential in cancer development (Karreth and Pandolfi, 2013; Sanchez-Mejias and Tay, 2015). Exploration of the mechanism in the ceRNA dysregulated process is therefore worthy of attention and may provide new insight into cancer diagnosis and treatment. In recent years, several researches had proved that the alternation of upstream factors had an impact on the downstream competitive relation between ceRNAs, which included somatic nucleotide variations (SNVs), copy number variations (CNVs), 3′UTR shortening, and transcription factors (TFs) (Li L. et al., 2014; Paci et al., 2014; Wang P. et al., 2020). Among them, Li et al. (2017) developed a comprehensive catalog and identified genetic variants that might be responsible for ceRNA dysregulation at the post-transcriptional level in human genome. Moreover, evidence had shown that the shortening in the 3′UTR region of ceRNA molecular could repress tumor-suppressor genes in trans in BRCA tissues (Park et al., 2018). However, whether another important upstream factor, DNA methylation, could disrupt ceRNA crosstalk is still unclear. Novel epigenetically diagnostic and prognostic biomarkers associated with ceRNA dysregulation should be further investigated.

In this study, we integrated the transcriptome expression and DNA methylation data to investigate the association between methylation and ceRNA dysregulation in multiple cancer types. Using modified mutual information-based method, we not only identified cancer or normal context-specific dysregulated lncRNA–mRNA interactions but also extracted triples with significant differences between both statuses. These data provided more comprehensive DysCeNets than those that care much about the cancer specificity dysregulated interactions. Through the topological properties analysis of each DysCeNet as well as the conservative attribute analysis at the pan-cancer level, it was found that lncRNAs played essential roles in the ceRNA dysregulation process. Furthermore, we illustrated the landscape of ER lncRNAs related to ceRNA dysregulation. The ER lncRNAs that occurred in single cancer type showed a regulatory tendency, while the pan-cancer ER lncRNAs were found to be affected in a complex pattern. We also investigated the attribute of interactions linked to ER lncRNAs and found that ER lncRNAs dominated vital positions in DysCeNets. Our study developed a novel strategy to interpret DNA methylation effect in ceRNA dysregulation and highlights the essential roles of ER lncRNAs in the cancer process.

It is important to determine the diagnostic and prognostic efficiency of pan-cancer ER lncRNAs since those lncRNAs were found to be associated with multiple cancer hallmarks. Multiple evidences have proved the capacity of ER lncRNAs in single cancer type. For instance, the transcriptional activity of EA lncRNA PVT1 was strongly upregulated and associated to promoter hypomethylation in KIRC (Posa et al., 2016), which was consistent with our result, and its misregulation could predict unfavorable prognosis in KIRC patients (Bao et al., 2017). As other examples, rs145204276 affected the methylation status of the EA lncRNA GAS5 promoter and subsequently upregulated its expression in Chinese HCC samples (Tao et al., 2015). Moreover, lncRNA GAS5 could promote tumor progression by targeting TP53INP1 in hepatocellular carcinoma (Zhang et al., 2019) and the GAS5/TP53INP1 axis was also identified as gain interaction in our LIHC DysCeNet. All these observations suggested the important roles of ER lncRNAs on carcinogenesis and tumor progression. Therefore, we developed a systematic strategy that considers both methylation status and expression level, and identified nine ER lncRNAs with pan-cancer diagnostic capacity. A recent study has proved that non-coding RNA could serve as a survival predictor of cancer (Bao et al., 2021). Therefore, the prognostic efficacy of the abovementioned ER lncRNAs had also been verified.

An increasing number of researches suggest that tumor microenvironment plays a crucial role in cancer therapy (Zhang Z. et al., 2020). As a critical immune regulator, lncRNA has been found to correlate with immune cell infiltration and immunotherapy response in different cancer types (Sun et al., 2020; Zhou et al., 2020). Therefore, we analyzed the relationship between pan-cancer ER lncRNAs and various immune cells through ImmLnc (Li Y. et al., 2020). We found that the expression of seven ER lncRNAs was significantly correlated with the immune cell infiltration (Supplementary Figure 6). Moreover, mostly ER lncRNAs showed the same correlation direction across multiple cancers. Regarding the high frequency of immune cell infiltration-related lncRNA HCP5, the lncRNA has been reported to sponge miR-150-5p and upregulated the expression of PD-L1/CD274, thus promoting tumor growth and affecting immunotherapy (Xu et al., 2020). These results suggest a potential role among DNA methylation, ceRNA mechanism, and immune regulation, and lncRNAs may be the key molecules in this process.

## Conclusion

In summary, this work integrated multi-dimensional data to reconstruct the dysregulated ceRNA networks across eight cancer types and focused on lncRNA as the entry point to identify ER lncRNA that was involved in ceRNA dysregulation. The influence of the ER lncRNA on ceRNA dysregulation was deeply explored, and the possibility of the ER lncRNA as cancer diagnostic and prognostic biomarkers was verified. Along with the exploration on the relationship between ceRNA dysregulation and upstream regulators, this study will provide a novel insight for understanding the impact of DNA methylation on the post-transcriptional regulation and promote epigenetics research in cancer tumorigenesis and progression.

## Data Availability Statement

The original contributions presented in the study are included in the article/Supplementary Material, further inquiries can be directed to the corresponding author/s.

## Author Contributions

KL, JL, and HW conceived and designed the experiments. DX, LW, SP, MC, WW, XY, ZX, and JX analyzed the data. KL and JL wrote the manuscript. All authors have read and approved the final manuscript.

## Conflict of Interest

The authors declare that the research was conducted in the absence of any commercial or financial relationships that could be construed as a potential conflict of interest.

## Footnotes

1 https://cancergenome.nih.gov/

2 www.ncbi.nlm.nih.gov/geo/
