# Supplementary material for: The Functional Characterization of Epigenetically Related lncRNAs Involved in Dysregulated CeRNA–CeRNA Networks Across Eight Cancer Types
Source: Front Cell Dev Biol. 2021 Jun 17;9:649755. doi: 10.3389/fcell.2021.649755 (PMC8247484; doi:10.3389/fcell.2021.649755)
Supplement: Supplementary file 1 [file Data_Sheet_1.PDF]

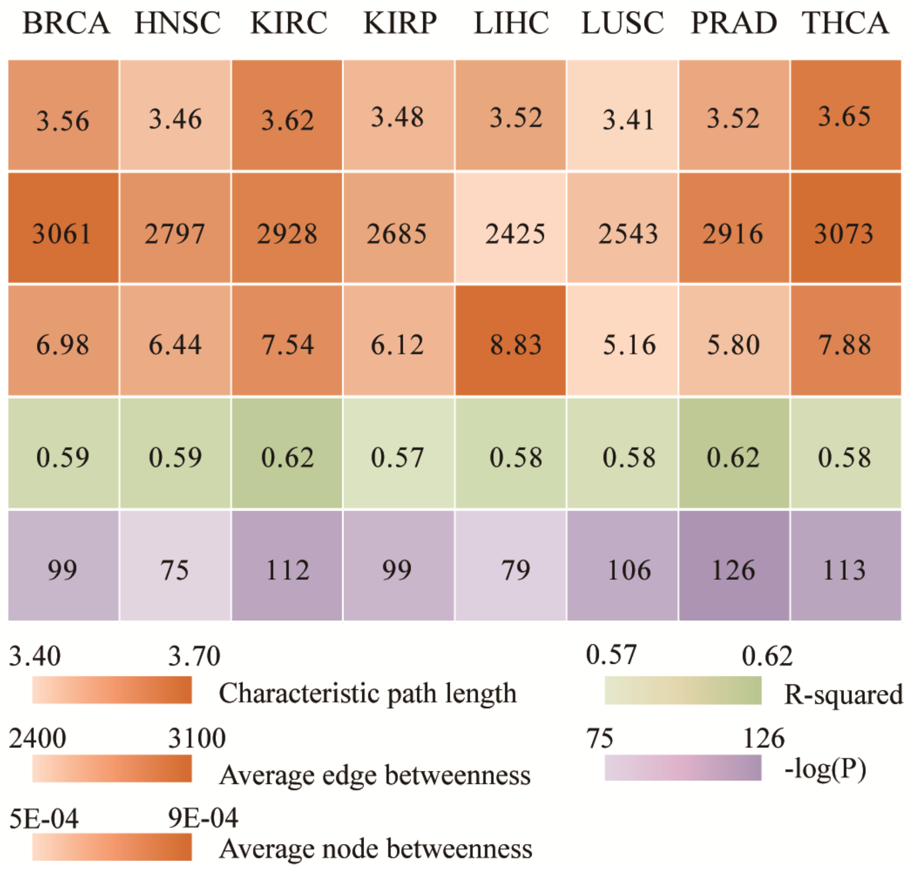

Figure S1. Properties feature of DysCeNets across eight cancer types. The first row represents the characteristic path length. And the second and the third rows represent the average edge and node betweenness. These attributes are significantly higher than random networks (All p-value < 0.001). The fourth indicates the goodness of fit of degree distribution. The fifth is the −log (p) of node degree difference between mRNA and lncRNA.

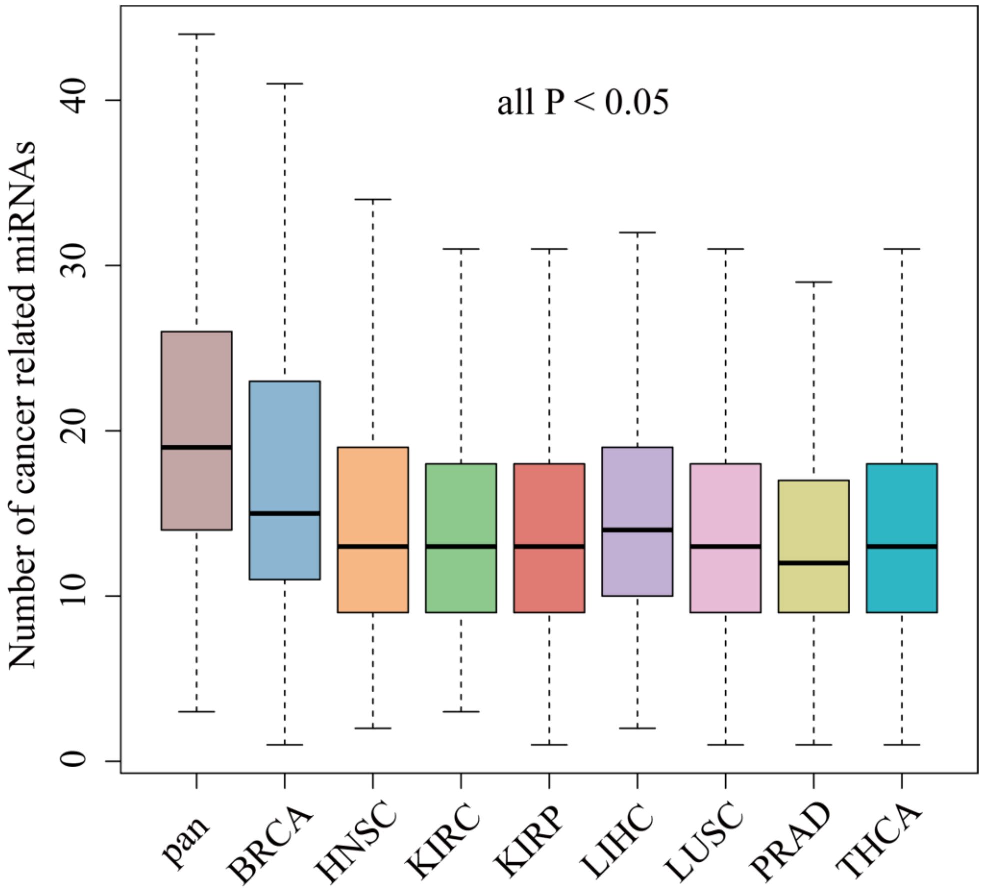

Figure S2. The comparison of the number of cancer-related miRNAs between the core component ceRNAs and single dysregulated network. The number of cancer-related miRNAs in core component was significantly increased than those in a single dysregulated network (Wilcoxon test, all P value < 0.05).

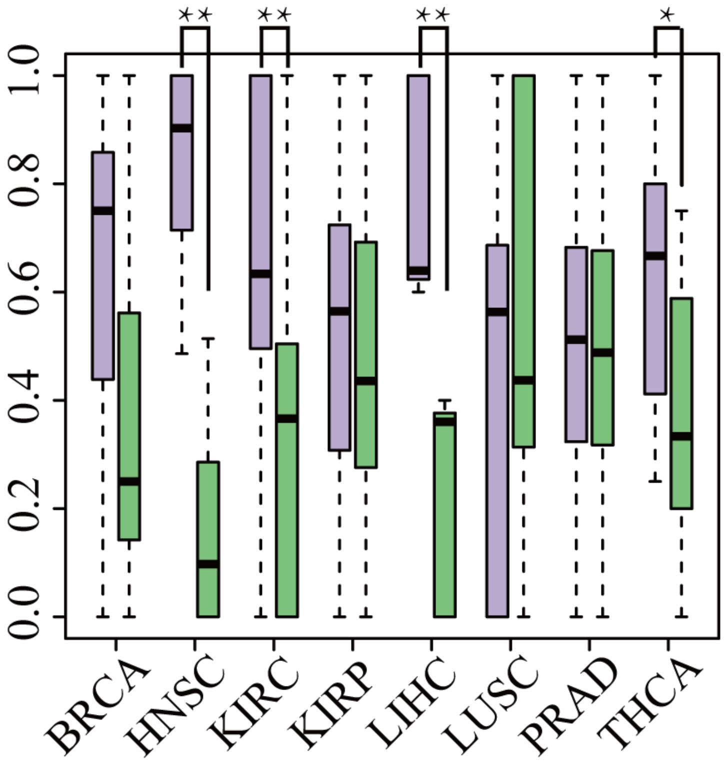

Figure S3. The property of edge linked to ER lncRNAs in DysCeNets. **: p value < 0.05, *: p value < 0.1.

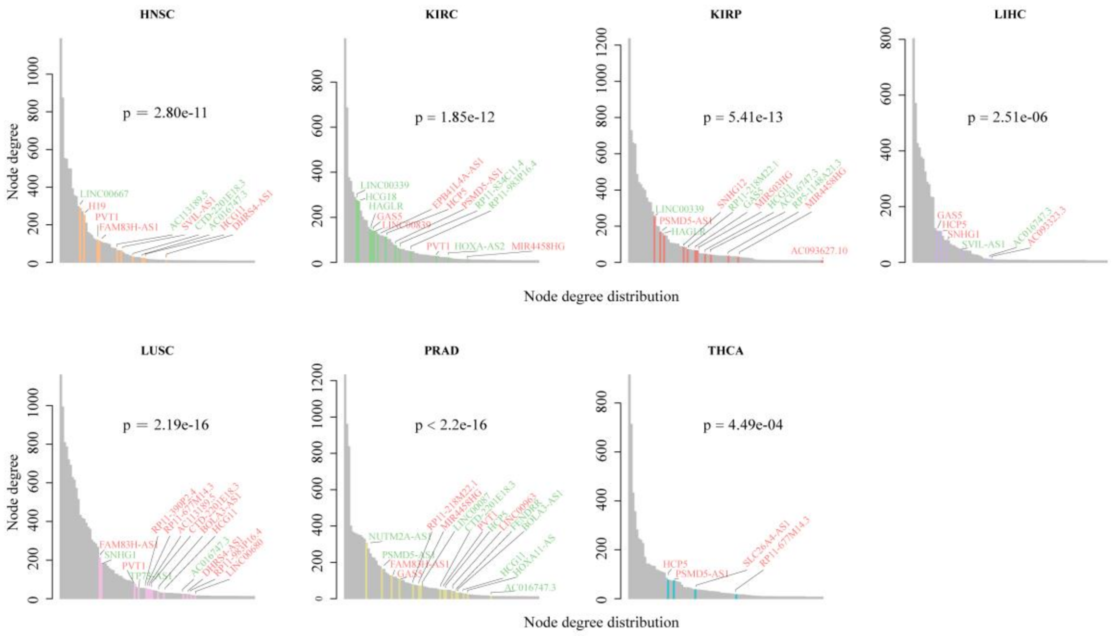

Figure S4. The node degree distribution of top 100 ceRNAs in each DCNet. The colored column represents the node degree of ER lncRNAs. The symbol colored red represents EA lncRNAs, the symbol colored green represents ES lncRNAs. All p-values were calculated using fisher test.

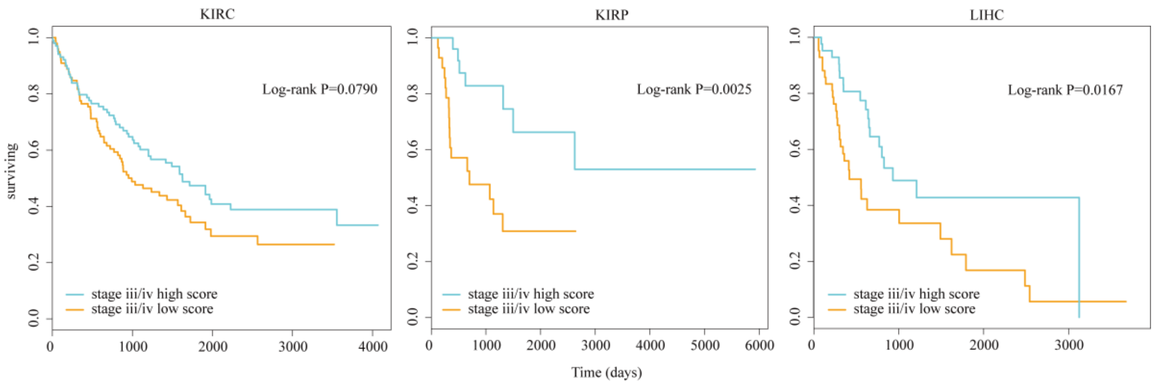

Figure S5. Kaplan-Meier estimates of the OS between low socre and high score group with high-stage patients according to the ER lncRNAs. The score was calculated based on KIRC expression, KIRP methylation and LIHC methylation levels.

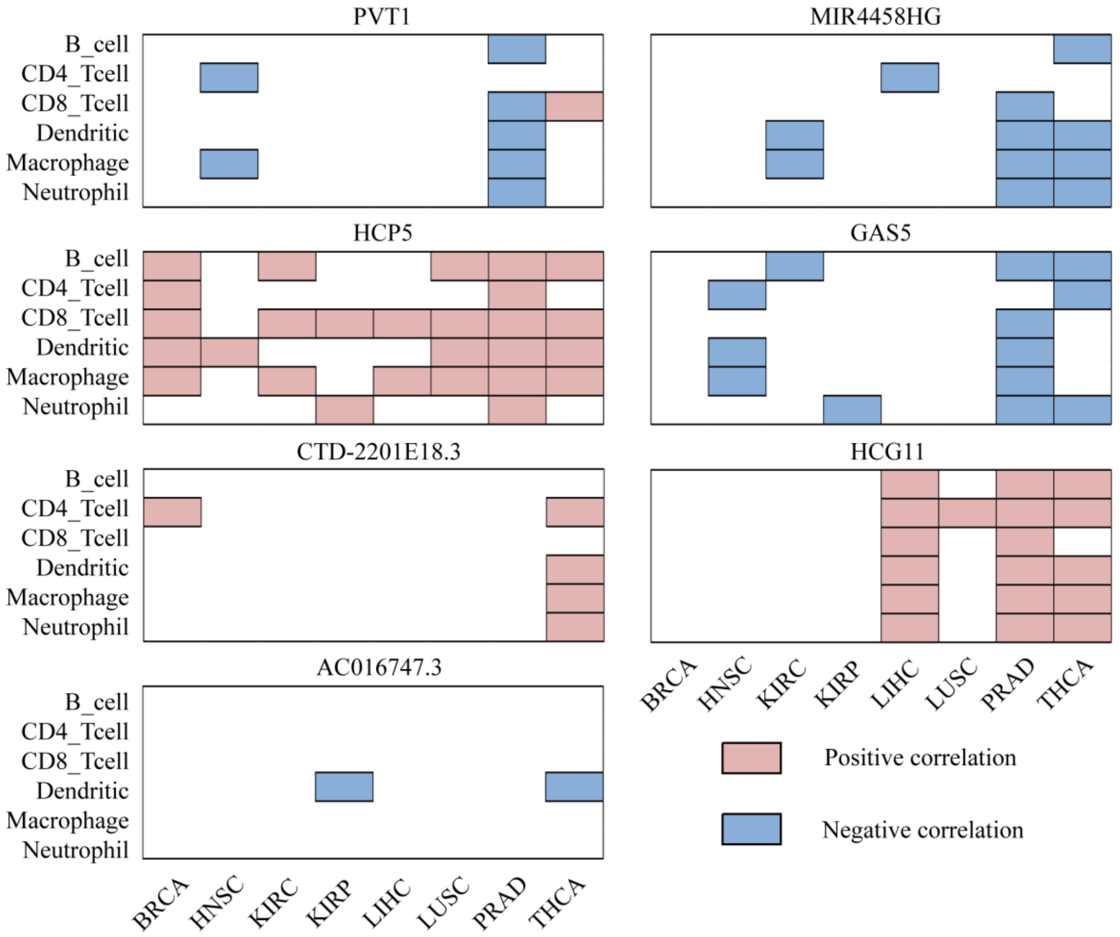

Figure S6. The correlation between the expression of ER lncRNAs and immune cell infiltration through ImmLnc. The red rectangle represent the lncRNA positively correlate with immune cell, the blue rectangle represent the lncRNA negatively correlate with immune cell.

Table S1. Number of samples in expression and methylation datasets across 8 cancer types from TCGA database

	Expression(mRNA/lncRNA)		Expression(miRNA)		Methylation	
	cancer	normal	cancer	normal	cancer	normal
BRCA	102	102	102	102	68	68
HNSC	43	43	42	42	43	20
KIRC	72	72	71	71	72	24
KIRP	31	31	31	31	31	22
LIHC	50	50	49	49	50	41
LUSC	49	49	38	38	49	7
PRAD	52	52	52	52	52	35
THCA	58	58	58	58	58	49

Table S2. Information of 15 independent datasets from GEO database

GEO id	cancer type	platform	type	cancer sample	normal sample
GSE42568	BRCA	GPL570	expression	104	17
GSE29330	HNSC	GPL570	expression	13	5
GSE36895	KIRC	GPL570	expression	29	23
GSE48352	KIRP	GPL16311	expression	24	8
GSE112790	LIHC	GPL570	expression	183	15
GSE18842	LUSC	GPL570	expression	46	45
GSE69223	PRAD	GPL570	expression	15	15
GSE60542	THCA	GPL570	expression	33	30
GSE66695	BRCA	GPL13534	methylation	79	40
GSE40005	HNSC	GPL13534	methylation	12	12
GSE61441	KIRC	GPL13534	methylation	46	46
GSE113019	LIHC	GPL13534	methylation	37	18
GSE56044	LUSC	GPL13534	methylation	23	12
GSE112047	PRAD	GPL13534	methylation	31	16
GSE86961	THCA	GPL13534	methylation	41	41

Table S3. Identification of Epigenetically regulated lncRNAs (Epigenetically activated and Epigenetically silenced)

	HM450 probe type			Beta value	
Correlation	Normal	Cancer	<0.25	(0.25,0.75)	>0.75
SNC	CUN	CUT	NA	ES	ES
SNC	CUN	VMT	NA	ES	ES
SNC	CUN	IMT	NA	ES	ES
SNC	CUN	CMT	NA	ES	ES
SNC	VMN	CUT	EA	NA	ES
SNC	VMN	VMT	EA	NA	ES
SNC	VMN	IMT	EA	NA	ES
SNC	VMN	CMT	EA	NA	ES
SNC	IMN	CUT	EA	NA	ES
SNC	IMN	VMT	EA	NA	ES
SNC	IMN	IMT	EA	NA	ES
SNC	IMN	CMT	EA	NA	ES
SNC	CMN	CUT	EA	EA	NA
SNC	CMN	VMT	EA	EA	NA
SNC	CMN	IMT	EA	EA	NA
SNC	CMN	CMT	EA	EA	NA
WNC	CUN	CUT	NA	NA	NA
WNC	CUN	VMT	NA	ES	ES
WNC	CUN	IMT	NA	ES	ES
WNC	CUN	CMT	NA	ES	ES
WNC	VMN	CUT	NA	NA	NA
WNC	VMN	VMT	NA	NA	NA
WNC	VMN	IMT	NA	NA	NA
WNC	VMN	CMT	NA	NA	NA
WNC	IMN	CUT	NA	NA	NA
WNC	IMN	VMT	NA	NA	NA
WNC	IMN	IMT	NA	NA	NA
WNC	IMN	CMT	NA	NA	NA
WNC	CMN	CUT	EA	NA	NA
WNC	CMN	VMT	EA	NA	NA
WNC	CMN	IMT	EA	NA	NA
WNC	CMN	CMT	NA	NA	NA
NNC	CUN	CUT	NA	NA	NA
NNC	CUN	VMT	NA	NA	NA
NNC	CUN	IMT	NA	NA	NA
NNC	CUN	CMT	NA	NA	NA
NNC	VMN	CUT	NA	NA	NA
NNC	VMN	VMT	NA	NA	NA
NNC	VMN	IMT	NA	NA	NA

NNC	VMN	CMT	NA	NA	NA
NNC	IMN	CUT	NA	NA	NA
NNC	IMN	VMT	NA	NA	NA
NNC	IMN	IMT	NA	NA	NA
NNC	IMN	CMT	NA	NA	NA
NNC	CMN	CUT	NA	NA	NA
NNC	CMN	VMT	NA	NA	NA
NNC	CMN	IMT	NA	NA	NA

EA: Epigenetic Activation, ES: Epigenetic Silencing, NA: other status not considered

Table S4. The information of ER lncRNAs occurred in single cancer type

Cancer types	EA lncRNAs	ES lncRNAs	Multi-ER lncRNAs
BRCA	H19, TP73-AS1, BOLA3-AS1, PVT1, FAM83H-AS1, AC093323.3, PSMD5-AS1	HCG11, CTD-2201E18.3, RP11-834C11.4, DHRS4-AS1, AC016747.3, MIR497HG, NUTM2A-AS1, SNHG3	-
HNSC	PVT1, FAM83H-AS1, DHRS4-AS1, HCG11	H19, AC113189.5, CTD-2201E18.3, AC016747.3, LINC00667	SVIL-AS1
KIRC	GAS5, PVT1, HCP5, EPB41L4A-AS1, LINC00839, MIR4458HG, PSMD5-AS1	C1RL-AS1, HCG11, HOXA-AS2, HCG18, ZNF790-AS1, RP11-834C11.4, RP11-983P16.4, MAGI2-AS3, LINC00339, DHRS4-AS1, HAGLR	-
KIRP	HOXA-AS2, MIR503HG, AC093627.10, MIR4458HG, PSMD5-AS1	SNHG12, GAS5, MAGI2-AS3, RP11-218M22.1, LINC00339, AC016747.3, HAGLR, RP5-1148A21.3, HCG11	-
LIHC	GAS5, SNHG1, HCP5, JPX, AC093323.3	AC016747.3, SVIL-AS1	-
LUSC	LOXL1-AS1, AC113189.5, CTD-2201E18.3, BOLA3-AS1, RP11-983P16.4, PVT1, MAGI2-AS3, FAM83H-AS1, DHRS4-AS1, LINC00680, RP11-390P2.4, HCG11	RP11-677M14.3, SNHG1, TP73-AS1, AC016747.3	-
PRAD	GAS5, PVT1, FAM83H-AS1, LINC00963, MIR4458HG	CTD-2201E18.3, HOXA11-AS, LINC00087, FENDRR, BOLA3-AS1, HCP5, AC016747.3, NUTM2A-AS1, HCG11, PSMD5-AS1	RP11-218M22.1
THCA	RP11-677M14.3, HCP5, LINC00152, LINC00680, AC005083.1, MIR4458HG, PSMD5-AS1	SLC26A4-AS1, AC113189.5	-

Table S5. The information of ER lncRNAs occurred in pan-cancer type

lncRNA	Epigenetic status	EA cancer types	ES cancer types	Multi-ER cancer types
PVT1	EA	BRCA, HNSC, KIRC, LUSC, PRAD	-	-
FAM83H-AS1	EA	BRCA, HNSC, LUSC, PRAD	-	-
AC093323.3	EA	BRCA, LIHC	-	-
PSMD5-AS1	EA	BRCA, KIRC, KIRP, THCA	PRAD	-
GAS5	EA	KIRC, LIHC, PRAD	KIRP	-
HCP5	EA	KIRC, LIHC, THCA	PRAD	-
MIR4458HG	EA	KIRC, KIRP, PRAD, THCA	-	-
LINC00680	EA	LUSC, THCA	-	-
CTD-2201E18.3	ES	LUSC	BRCA, HNSC, PRAD	-
RP11-834C11.4	ES	-	BRCA, KIRC	-
AC016747.3	ES	-	BRCA, HNSC, KIRP, LIHC, LUSC, PRAD	-
NUTM2A-AS1	ES	-	BRCA, PRAD	-
LINC00339	ES	-	KIRC, KIRP	-
HAGLR	ES	-	KIRC, KIRP	-
DHRS4-AS1	Multi	HNSC, LUSC	BRCA, KIRC	-
SVIL-AS1	Multi	-	LIHC	HNSC
AC113189.5	Multi	LUSC	HNSC, THCA	-
HCG11	Multi	HNSC, LUSC	BRCA, KIRC, KIRP, PRAD	-
HOXA-AS2	Multi	KIRP	KIRC	-
RP11-983P16.4	Multi	LUSC	KIRC	-
MAGI2-AS3	Multi	LUSC	KIRC, KIRP	-
RP11-218M22.1	Multi	-	KIRP	PRAD
SNHG1	Multi	LIHC	LUSC	-
RP11-677M14.3	Multi	THCA	LUSC	-
H19	Multi	BRCA	HNSC	-
TP73-AS1	Multi	BRCA	LUSC	-
BOLA3-AS1	Multi	BRCA, LUSC	PRAD	-

Table S6. Statistics of lncRNAs characteristics in the ceRNA networks compared with lncRNAs not involved in the ceRNA networks

	lncRNA_IN	lncRNA_OUT	length_p	exon_p	exp_p
BRCA	101	6858	5.55E-06	3.26E-12	2.21E-57
HNSC	69	5332	4.20E-03	5.42E-07	1.50E-42
KIRC	95	6808	1.14E-05	4.93E-15	7.91E-52
KIRP	101	6607	1.42E-05	5.70E-14	5.55E-53
LIHC	51	4548	1.71E-02	3.27E-06	6.47E-32
LUSC	96	6995	4.81E-05	1.85E-11	3.50E-55
PRAD	97	6713	2.56E-06	1.19E-11	5.41E-55
THCA	102	6906	7.38E-06	2.64E-11	9.01E-56

Table S7. The AUC of the ROC curves based on 9 ER lncRNAs

	exp	methy	Exp+methy	Ce_lnc	Array_lnc	GEO_exp	GEO_methy
BRCA	0.8133	0.9415	0.8817	0.5795	0.5860	0.8710	0.8881
HNSC	0.4965	0.3988	0.7412	0.5877	0.5783	0.8077	0.9826
KIRC	0.3052	0.9795	0.9592	0.6196	0.6197	0.7916	0.7124
KIRP	0.3564	0.8710	0.8330	0.6169	0.6394	0.6510	NA
LIHC	0.4526	0.9115	0.8922	0.6181	0.6160	0.6645	0.9459
LUSC	0.8884	0.9898	0.9917	0.6090	0.6183	0.7094	1.0000
PRAD	0.7663	0.7302	0.8558	0.6062	0.6046	0.7667	0.8246
THCA	0.2628	0.8637	0.8832	0.5851	0.6076	0.7227	0.8685
